# Layer-specific potentiation of network GABAergic inhibition in the CA1 area of the hippocampus

**DOI:** 10.1038/srep28454

**Published:** 2016-06-27

**Authors:** Michelangelo Colavita, Geoffrey Terral, Clement E. Lemercier, Filippo Drago, Giovanni Marsicano, Federico Massa

**Affiliations:** 1 INSERM U1215, NeuroCentre Magendie, AVENIR Group “Endocannabinoids and Neuroadaptation”, 33077 Bordeaux, France; 2 Université de Bordeaux, 33077 Bordeaux, France; 3 University of Catania, Biometec – Department of Biomedical and Biotechnological Sciences, 95125 Catania, Italy

## Abstract

One of the most important functions of GABAergic inhibition in cortical regions is the tight control of spatiotemporal activity of principal neuronal ensembles. However, electrophysiological recordings do not provide sufficient spatial information to determine the spatiotemporal properties of inhibitory plasticity. Using Voltage Sensitive Dye Imaging (VSDI) in mouse hippocampal slices, we demonstrate that GABA_A_-mediated field inhibitory postsynaptic potentials undergo layer-specific potentiation upon activation of metabotropic glutamate receptors (mGlu). VSDI recordings allowed detection of pharmacologically isolated GABA_A_-dependent hyperpolarization signals. Bath-application of the selective group-I mGlu receptor agonist, (S)-3,5-Dihydroxyphenylglycine (DHPG), induces an enhancement of the GABAergic VSDI-recorded signal, which is more or less pronounced in different hippocampal layers. This potentiation is mediated by mGlu_5_ and downstream activation of IP_3_ receptors. Our results depict network GABAergic activity in the hippocampal CA1 region and its sub-layers, showing also a novel form of inhibitory synaptic plasticity tightly coupled to glutamatergic activity.

In the mammalian brain, the main source of inhibition is provided by the neurotransmitter gamma-aminobutyric acid (GABA), which acts on two classes of receptors: the ionotropic GABA_A_ and the metabotropic GABA_B_^1^.

In cortical areas, GABA is released by locally projecting interneurons, which are estimated to account for approximately 11% of the total cell population in the hippocampal CA1 region^2^,^3^. However, despite the paucity of these cells within this region, each interneuron can make synapses with several hundreds of pyramidal cells^4^ and other interneurons^4^,^5^, providing an extremely complex and powerful spatiotemporal control of network activity.

At least 21 different classes of interneurons have been described in the CA1, classified on the basis of firing patterns, molecular expression profiles, and innervation properties^4^,^6^,^7^. This high morpho-physiological heterogeneity, together with the high degree of synaptic connectivity between pyramidal cells and other interneurons, suggest the existence of a “network of interneurons” with a key role in controlling hippocampal computations^5^,^8^,^9^. For instance, GABAergic cells through the release of GABA and subsequent activation of GABA_A_ receptors hyperpolarize pyramidal cells^10^. Thus, depending on the wiring scheme of interneurons onto principal cells, feedback and/or feed forward inhibition may occur, which are fundamental processes in shaping the spatial and temporal profile of principal cell firing and global network activity^11^,^12^,^13^.

Moreover, the existence of GABAergic synapses between different types of interneurons^14^,^15^, including specialized interneuron-specific cells^4^,^5^, suggests that the inhibitory control of other interneurons is crucial in providing a higher level of coordination of hippocampal network activity^5^.

Due to technical limitations, such as the difficulty to obtain reliable electrophysiological recordings of local “inhibitory fields” by standard electrophysiological approaches^16^,^17^,^18^, very little is known concerning the global network activity and dynamics of interneurons. Indeed, powerful single-cell recordings, widely used to study the roles of inhibitory activity at the single cell level, is not appropriate to observe the global spatiotemporal patterns of activity of inhibitory networks. Such a “mesoscopic” level of analysis of local inhibitory systems is, therefore, a lacking element in the quest for understanding dynamics and properties of principal networks.

Voltage sensitive dye imaging (VSDI) allows coincident optical monitoring of neuronal activity within a wide range of spatial resolution (from very small cell compartments such as dendrites, to areas of several mm^2^), at millisecond time scale^19^,^20^. After binding cell membranes, voltage-sensitive dye molecules emit fluorescence proportionally to changes in membrane potential^21^. VSDI has been widely used to study excitatory network activity and single cell properties of neurons^22^,^23^,^24^. Notably, at regional (mesoscopic) level, VSDI allows for the dissection of depolarization signals within different anatomical compartments (e.g., hippocampal sub-layers). However, this feature of VSDI has not yet been used to characterize, pharmacologically and anatomically, the activity and plasticity of inhibitory networks.

Group-I metabotropic glutamate receptors (group-I mGlu receptors) include mGlu_1_ and mGlu_5_^25^. They constitute a subclass of metabotropic glutamate receptors that are coupled to G_q_ heterotrimeric G proteins, thus leading to activation of phospholipase C and subsequent mobilization of inositol 1,4,5-trisphosphate (IP_3_), which in turn increases cytosolic Ca^2+^ *via* activation of IP_3_ receptors on the endoplasmic reticulum^26^. Activation of group-I mGlu receptors is known to strongly impact on synaptic properties and plasticity of hippocampal circuits^27^,^28^.

In this study, we took advantage of the VSDI technique to visualize and quantify evoked field inhibitory postsynaptic potentials (fIPSPs) in the CA1 hippocampal region, and to analyze their temporal and spatial features within the different sub-layers. In addition, we found that activation of mGlu_5_ leads to an IP_3_-dependent potentiation of fIPSPs in a sub-region specific manner. These findings demonstrate the spatial and temporal distribution of GABAergic activity in the CA1 region of hippocampus and, most importantly, they show that metabotropic glutamatergic signaling bears a strong impact on the global and local activity of inhibitory networks in specific brain regions.

## Results

### GABA_A_-mediated network activity in the hippocampal CA1

**Figure 1:**
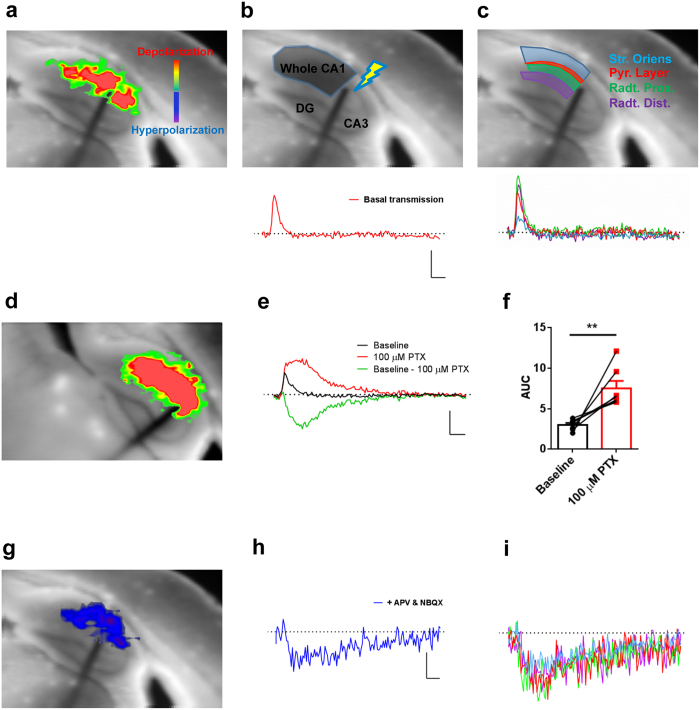
Blockade of ionotropic glutamatergic transmission reveals network hyperpolarization throughout CA1. **(a**) Representative frame showing VSDI-recorded depolarization activity after stimulation of Schaffer’s collateral in drug-free ACSF. Inset color scale indicates depolarization (green to red) and hyperpolarization (blue to purple). **(b,c**) Representative regions of interest (ROIs) arrangement (upper panels) and corresponding traces (lower panels) showing resulting average VSDI-recorded depolarization in drug-free ACSF from a ROI covering the whole CA1 (**b**) and in its different sub-regions (**c**). Str. Oriens = *stratum oriens*, Pyr. Layer = Pyramidal Layer, Radt. Prox. = *radiatum* proximal, Radt. Dist. = *radiatum* distal. **(d**) Representative frame showing VSDI-recorded depolarization activity after stimulation of Schaffer’s collateral following application of Picrotoxin (PTX, 100 μM). **(e**) Representative traces showing the impact of GABAergic transmission on VSDI-recorded depolarization from a ROI covering the whole CA1 before (Baseline) and after application of PTX. The GABAergic component (Baseline – 100 μM Picrotoxin) is obtained from the difference between Baseline and PTX conditions, respectively. **(f**) Quantification through area under curve (AUC) calculation of VSDI-recorded depolarization in the whole CA1 before (Baseline) and after application of PTX. n = 7 slices from 5 mice. Bars represent mean ± s.e.m. whereas over imposed lines are single values. **p < 0.01, two-tailed paired *t*-test. **(g)** Representative frame showing VSDI-recorded hyperpolarization activity after stimulation of Schaffer’s collateral in presence of blockers of ionotropic glutamatergic transmission (APV & NBQX). **(h,i**) Representative traces showing VSDI-recorded hyperpolarization activity following APV & NBQX application from a ROI covering the whole CA1 (**h**) and in its different sub-regions (**i**). Scale bars are 25 milliseconds on X-axis and 0.2% ΔF*F^−1^ on y-axis for traces in (**b,c,e**) whereas 0.05% ΔF*F^−1^ on y-axis for traces in (**h,i**).

Stimulation of Schaffer’s collateral pathway in hippocampal slices stained with Di-4-ANEPPS in drug-free ACSF produces a depolarization spanning along the horizontal axis of CA1 (Fig. 1a; green to red color-scale; Supplementary Video S1). To quantify depolarization-mediated VSDI signal, we drew a region of interest (ROI) covering the whole CA1 (Fig. 1b) and the resulting mean ΔF^*^F^−1^ values over time are shown as an upward deflection of the signal lasting approximately 30 milliseconds (Fig. 1b, trace). More detailed analysis of VSDI-recorded depolarization revealed detectable signal specifically in the different layers of CA1, due to action potentials spreading along pyramidal cells (Fig. 1c, representatives ROIs arrangement and corresponding traces). Consistent with previous reports under similar conditions^23^,^29^, these images represent the spreading of depolarization signals within the CA1 region. Next, we examined whether a GABAergic component could be identified in these VSDI recordings. Application of the GABA_A_ receptor antagonist Picrotoxin (100 μM) induced an increase in the intensity of evoked depolarization signals both in the whole CA1 (Fig. 1d–f, Supplementary Video S2; AUC: baseline, 3 ± 0.2; PTX, 7.5 ± 2.4; p = 0.0068 baseline *vs* PTX, paired *t* test) as well as in the different sub-fields (Supplementary Fig. S1). Thus, VSDI depolarization signals result from the simultaneous activation of excitatory and inhibitory networks. To study in detail this GABAergic component of network activity, we isolated inhibitory neurotransmission by applying a cocktail of AMPA/Kainate and NMDA receptors antagonists (NBQX 10 μM and APV 50 μM respectively). This treatment fully abolished the depolarization signals in the whole CA1 and each CA1 sub-region (Fig. 1g–i; Supplementary Video S3), confirming their glutamatergic ionotropic origins. Importantly, however, blockade of ionotropic glutamatergic receptors also revealed a clear downward deflection of the traces below background fluorescence levels, which lasted approximately 200–250 milliseconds and was compatible with a hyperpolarizing event (Fig. 1g–i; blue color scale; Supplementary Video S3). Thus, stimulation of Schaffer’s collaterals induces reliable and quantifiable hyperpolarizing field signals (hereafter called fIPSPs, i.e. field inhibitory postsynaptic potentials) in the CA1 region of the hippocampus.

**Figure 2:**
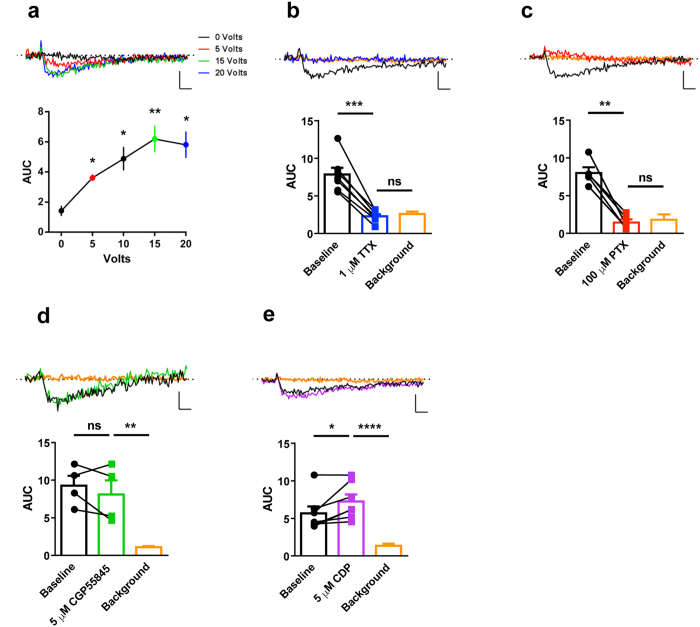
Characterization of the VSDI-recorded fIPSPs. **(a**) Increasing stimulation intensity every 5 minutes time steps significantly enhances the signal compared to 0 Volts [lower panel, one-way ANOVA with repeated measures over stimulation intensities (F_(2,605,13,02)_ = 12,95, p = 0.0005) followed by Tukey *post-hoc* test. n = 6 slices from 5 mice]. **(b)** 1 μM Tetrodotoxin (TTX) significantly abolishes the signal, which is statistically not different from the background, lower panel, n = 7 slices from 5 mice. **(c)** 100 μM Picrotoxin (PTX) significantly abolishes the signal, which is then not different from background, lower panel, n = 5 slices from 3 mice. **(d**) 5 μM CGP55845 does not affect the hyperpolarization signal, lower panel, n = 4 slices from 2 mice. **(e**) 5 μM Chlordiazepoxide (CDP) significantly increases the signal, lower panel, n = 7 slices from 4 mice. In all graphs, signal of interest has been assessed in a ROI covering the whole CA1. In all figures, traces in upper panels are the average of traces from each experimental condition. Bars (**b–e**) and points (**a**) represent mean ± s.e.m. whereas over imposed lines (**b–e**) are single values. Statistical significance has been assessed with two-tailed paired *t*-test between baseline condition and drug application, while two-tailed unpaired *t*-test has been used between drug application and respective background (**b–e**). Scale bars are 25 milliseconds on X-axis and 0.05% ΔF*F^−1^ on Y-axis. *p <0.05, **p < 0.01, ***p < 0.001, ****p < 0.0001, ns = not significant.

Next, we set to characterize the nature of these hyperpolarization signals. First, input/output experiments revealed that evoked fIPSPs depend on the intensity of the stimulation, reaching a plateau level at 15–20 Volts (Fig. 2a), suggesting that they rely on neuronal activity. The application of the voltage-gated Na^+^ channel blocker Tetrodotoxin (TTX, 1 μM) fully abolished fIPSPs in the whole CA1 (Fig. 2b, AUC: baseline, 7.8 ± 0.9; TTX, 2.3 ± 0.3; background, 2.6 ± 0.3; p = 0.0002 baseline *vs* TTX, paired *t* test; p = 0.5177 TTX vs background, unpaired *t* test) and in all hippocampal sub-regions (Supplementary Fig. S2) reducing them to basal background levels (see Methods for background definition). Thus, VSDI-recorded fIPSPs are not due to artifacts and depend on neuronal activity.

The main neurotransmitter mediating hyperpolarization in the brain is GABA, acting at ionotropic GABA_A_ or metabotropic GABA_B_ receptors^1^. Application of the GABA_A_ receptor antagonist Picrotoxin (PTX, 100 μM) abolished fIPSPs signal in the whole CA1 region (Fig. 2c; AUC: baseline, 8.0 ± 0.7; PTX, 1.4 ± 0.4; background, 1.8 ± 0.7; p = 0.0014 baseline *vs* PTX, paired *t* test; p = 0.6610 PTX vs background, unpaired *t* test). Conversely, the GABA_B_ receptor antagonist CGP55845 (5 μM) did not significantly alter CA1 fIPSPs (Fig. 2d; AUC: baseline, 9.3 ± 1.3; CGP55845, 8.1 ± 1.9; background, 1.1 ± 0.1; p = 0.3545 baseline *vs* CGP55845, paired *t* test; p = 0.01 CGP55845 vs background, unpaired *t* test), suggesting a specific involvement of GABA_A_ receptors in the observed network hyperpolarization. This was further confirmed by the application of the positive allosteric modulator of GABA_A_ receptor Chlordiazepoxide (CDP, 5 μM), which slightly but significantly increased fIPSPs amplitudes (Fig. 2e; AUC: baseline, 5.7 ± 0.9; CDP, 7.3 ± 0.9; background, 1.3 ± 0.3; p = 0.0320 Baseline *vs* CDP, paired *t* test; p < 0.0001 CDP vs Background, unpaired *t* test). Importantly, similar results were obtained when specific CA1 sub-regions were analyzed (Supplementary Fig. S2), further confirming the reliable nature of the observed VSDI signals as GABA_A_ receptor-dependent synaptic hyperpolarization events. Thus, VSDI allows detection and quantification of activity-dependent hyperpolarization events in the CA1 hippocampal region after stimulation of a large network of GABAergic interneurons.

### Spatial distribution of network GABA_A_ - mediated optical signals

**Figure 3:**
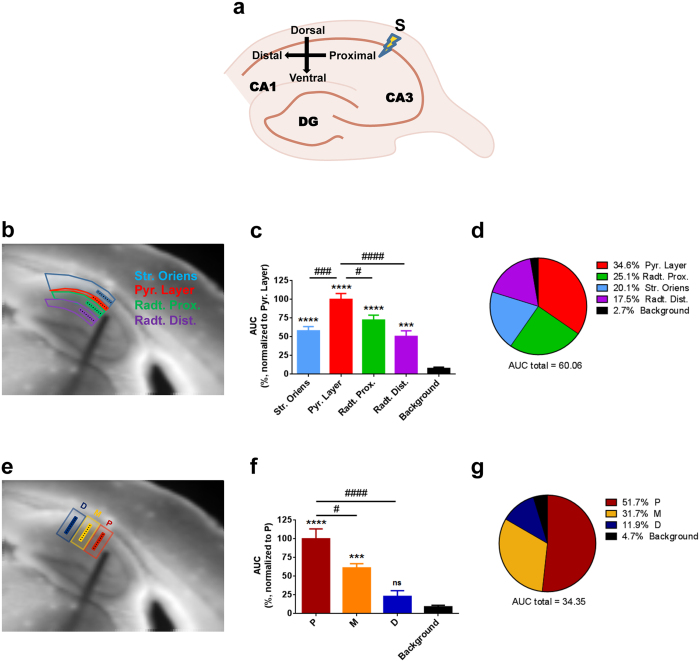
Spatial distribution of VSDI-recorded fIPSPs throughout CA1 region. **(a**) Cartoon representing the orientation of the different sub-regions studied in the CA1 (S, stimulation electrode). **(b**) Scheme showing spatial organization of the 8 pixels long ROI lines inside the dorso-ventral axis of CA1 (reference CA1 sub-regions are delimited by the colored contour). **(c**) Quantification through AUC of hyperpolarization from the ROI lines shows detectable signal in all the dorso-ventral regions of CA1, which is mainly localized in the pyramidal layer (one-way ANOVA followed by Tukey *post-hoc* test; asterisks are differences *vs.* “Background”, while hashes are differences *vs.* “Pyramidal layer”). **(d)** Pie chart summarizing the distribution of the signal from the lines along the dorso-ventral part of CA1 showing a predominant presence in the pyramidal layer (percentage of each region respect to the total AUC). **(e)** Scheme showing spatial organization of the 8 pixels long ROI lines inside the proximo-distal axis of CA1 (reference proximo-distal ROIs are delimited by the colored contour); “P” = proximal, “M” = medial, “D” = distal; distances from stimulation electrode are ~106, ~318 and ~531 μm for regions P, M and D respectively. **(f**) Quantification through AUC of hyperpolarization from the ROI lines shows detectable signal at the proximity of the stimulation electrode (lines in regions “P” and “M”) while signal from lines in region “D” is not different from background (one-way ANOVA followed by Tukey *post-hoc* test; asterisks are differences *vs.* “Background”, while hashes are differences *vs.* “P”). **(g)** Pie chart summarizing how the signal from the ROI lines along the proximo-distal part of CA1 is concentrated in the proximity of the stimulation electrode (percentage of each region respect to the total AUC). n = 10 slices from 10 mice. Data are mean ± s.e.m. #p < 0.05, ### and ***p < 0.001, #### and ****p < 0.0001, ns = not significant.

Unlike to classical electrophysiological recordings, the VSDI technique allows simultaneously observing synaptic events in different sub-regions of the area under investigation. Thus, we next quantified how the hyperpolarization signals induced by electrode stimulation are distributed among different CA1 sub-regions. Quantification of activity in equal ROIs distributed along the dorso-ventral axis of the CA1 region (Fig. 3a,b, see Methods) revealed that the strongest hyperpolarization was present in the CA1 pyramidal layer, whereas the *strata oriens* and *radiatum* (proximal and distal) displayed signals of lower amplitude (Fig. 3c,d). This observation is consistent with the fact that the majority of GABAergic synapses are located in the perisomatic area of CA1 pyramidal cells^2^,^30^.

Conversely, the intensity of the hyperpolarization signals decreased along the proximo-distal axis of CA1 (Fig. 3a,e–g), becoming undistinguishable from background levels at the most distal observed area (Fig. 3f,g), which were therefore excluded from further evaluations. These data indicate that the stimulation induces significant activation of the CA1 inhibitory network up to a distance of approximately 300–400 μm relative to the stimulation electrode, consistent with previous data obtained by single cell recordings^31^. Thus, electrical stimulation in the Schaffer’s collaterals region can activate a large population of CA1 interneurons, of which a relative majority appears to form perisomatic innervation of pyramidal cells.

### Group-I mGlu receptors activation potentiates network hyperpolarization

Group-I mGlu receptors have profound impact on neuronal activity, both on glutamatergic and GABAergic transmission^27^. In particular, field electrophysiological recordings of excitatory postsynaptic potentials (fEPSPs) showed that activation of group-I mGlu receptors decreases network excitatory transmission in the hippocampus and several other brain regions^27^,^32^. The study of metabotropic glutamatergic signaling on GABAergic activity, however, has been limited to date to single-cell recording settings^33^,^34^,^35^,^36^,^37^,^38^,^39^,^40^, with no studies focusing on network inhibition.

**Figure 4:**
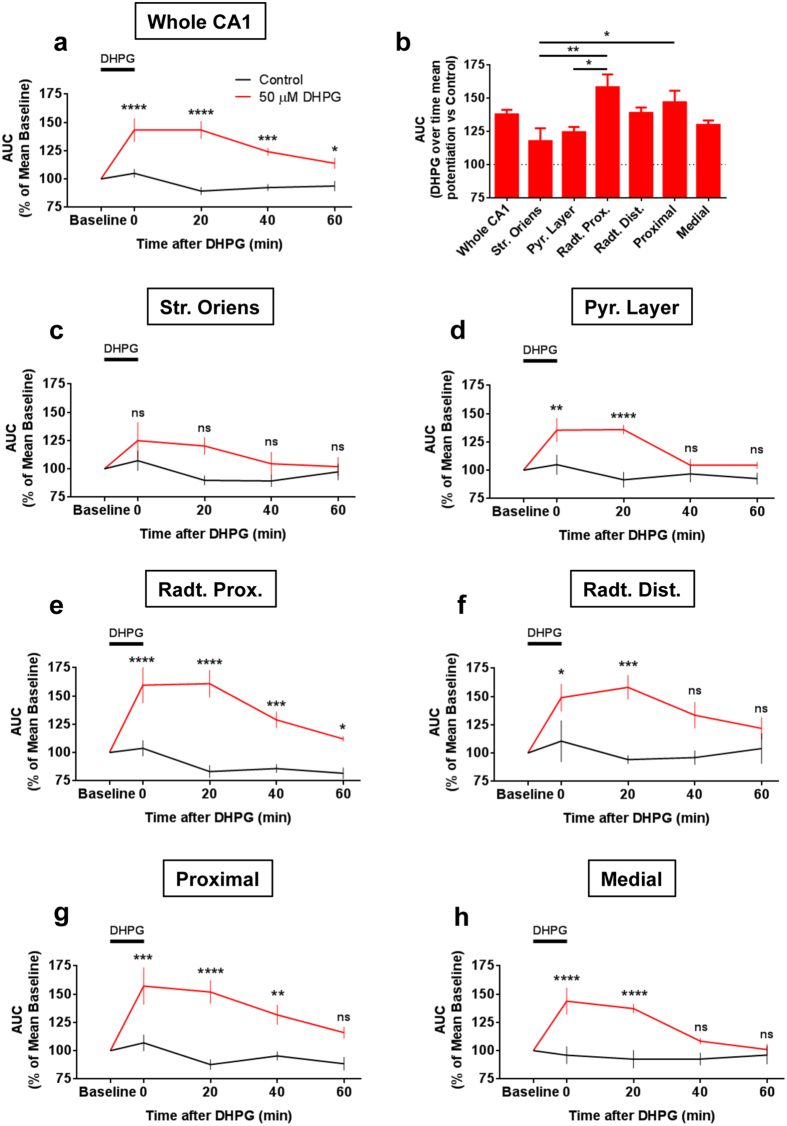
Long-lasting increase of VSDI-recorded fIPSPs after group-I mGlu receptors activation. Application of the selective group-I mGlu receptors agonist (S)-3,5-Dihydroxyphenylglycine (DHPG) for 10 minutes induces long-lasting enhancement of fIPSPs during washout of the drug in the whole CA1 **(a)** and in its different sub-regions **(c–h)**. **(b**) Graph showing mean over time percentage potentiation of DHPG *vs* control in whole CA1 and in its different sub-fields; one-way ANOVA followed by Tukey *post-hoc* test. n = (slices, mice): DHPG group = (7, 5), Control group = (7, 4). Data are mean ± s.e.m. Statistical analysis in (**a**) and (**c–h**) is two-way ANOVA followed by Bonferroni *post-hoc* test. *p < 0.05, **p < 0.01, ***p < 0.001, ****p < 0.0001, ns = not significant.

The application of the selective group-I mGlu receptor agonist (S)-3,5-Dihydroxyphenylglycine (DHPG, 50 μM, 10 minutes) led to a persistent enhancement of VSDI-recorded evoked fIPSPs in the whole CA1 region as compared to control conditions, which lasted beyond washout of the drug (Fig. 4a).

A closer dissection of the sub-regional fIPSPs distribution revealed that this effect of DHPG was present in different layers and in proximal and medial regions relative to the stimulation electrode (Fig. 4b). Interestingly, however, the DHPG effect differed in amplitude and duration in the different sub-regions analyzed. The magnitude was greatest in the proximal *stratum radiatum*, and minimal in the *stratum oriens* and pyramidal layer (Fig. 4b–h). Time-course analyses showed that the effect of DHPG lasted up to 60 minutes in the whole CA1 region (Fig. 4a), which was likely due to the impact of the proximal *stratum radiatum* (Fig. 4e). In contrast, the DHPG-induced potentiation of fIPSPs was of shorter duration in the pyramidal layer and distal *stratum radiatum* (Fig. 4d,f, 20 min). In the *stratum oriens*, two-way ANOVA analysis revealed a significant treatment effect (F _(1, 60)_ = 6.934, p = 0.0107), without “time x treatment” interaction, impeding the *post-hoc* determination of the time-dependent impact of DHPG (Fig. 4c). On the longitudinal axis, the amplitude of DHPG effect was not significantly different between areas located proximal or distal to the stimulation electrode (Fig. 4b). However, the DHPG-induced potentiation of fIPSPs was longer lasting in the CA1 portion closer to the electrode (Fig. 4g).

In addition, we tested if different doses and durations of DHPG application could also trigger a long-lasting increase in VSDI-recorded fIPSPs. As shown in Supplementary Fig. S3, DHPG application at half the previous concentration and for half the previous duration was sufficient to induce a persistent potentiation of network GABAergic activity in the whole CA1.

### mGlu_5_ mediates DHPG-induced potentiation of fIPSPs

**Figure 5:**
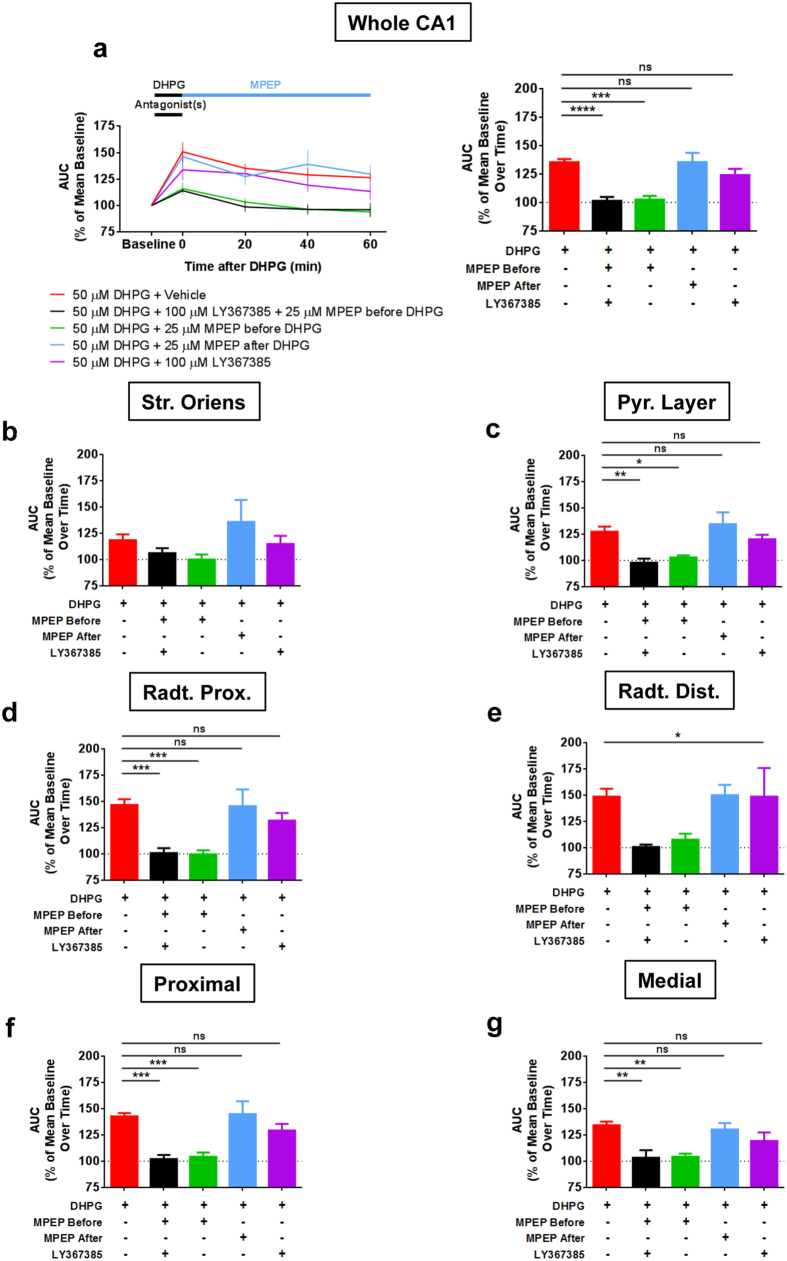
Long-lasting increase of fIPSPs after DHPG application is mediated by mGlu_5_ activation. Slices were incubated with DHPG in presence of antagonists of group-I mGlu receptors (LY367385 for mGlu_1_, MPEP for mGlu_5_ or both), which were kept until the end of DHPG application, except when MPEP has been applied after DHPG (represented in blue). In **(a)**, left panel shows time course, while bars in right panel represent averaged drug effects over time. In **(b–g)**, bars are the mean over time for each condition at the indicated region. One-way ANOVA followed by Dunnet *post-hoc* test has been used to assess differences between groups in (**a**) (right panel) and (**b–g**). In *radiatum* distal (**e**) there is a significant ANOVA (F_(4, 27)_ = 2.912, p = 0.04) but not significant Dunnet’s multiple comparisons test. n = (slices, mice): Vehicle group = (7, 7), LY367385 + MPEP before group = (7, 7), MPEP before group = (6, 5), MPEP after group (5, 4), LY367385 group = (7, 5). Data are mean ± s.e.m. **p < 0.01, ***p < 0.001, ****p < 0.0001, ns = not significant.

As DHPG activates both mGlu_1_ and mGlu_5_, we asked if either or both of these receptors are involved in the fIPSPs potentiation. Pretreatment of the slices with the specific mGlu_1_ antagonist LY367385 (100 μM) did not alter the effect of DHPG in the CA1 region (Fig. 5a). In contrast, the application of the specific mGlu_5_ antagonist MPEP (25 μM) fully blocked the DHPG-induced potentiation of fIPSPs (Fig. 5a). Importantly, when applied right after DHPG, MPEP was unable to block the potentiation of fIPSPs, showing that transient activation of mGlu_5_ receptors induces a genuine phenomenon of GABAergic synaptic plasticity, which is not due to lack of DHPG washout. Thus, activation of mGlu_5_ receptors is necessary for the induction but not for the maintenance of this plasticity.

At sub-regional level, similar results were obtained, with the exception of the *stratum oriens*, where, due to the weak effect of DHPG (see Fig. 4c), the data displays only non-significant trends (Fig. 5b). Thus, LY367385 did not alter the DHPG effect in any sub-region analyzed (Fig. 5c–g), whereas MPEP blocked this effect in all areas if applied before and not after DHPG (Fig. 5c–g). These data show that DHPG-induced potentiation of fIPSPs in different CA1 hippocampal sub-regions shares the same mechanisms, which rely on activation of mGlu_5_ receptors.

### Role of IP_3_ intracellular receptors

**Figure 6:**
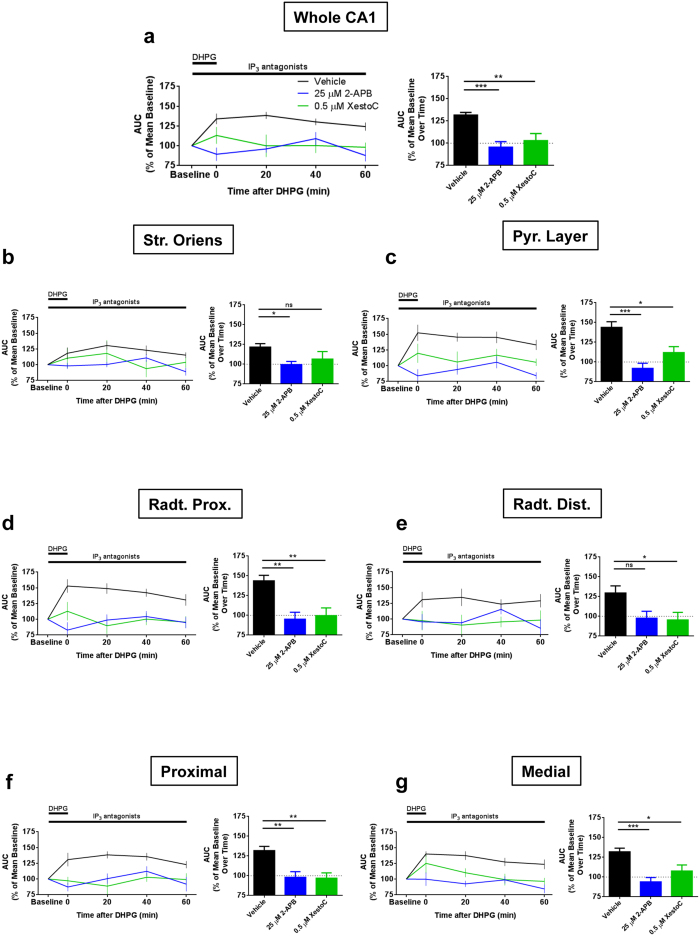
Long-lasting increase of fIPSPs after DHPG application is mediated by IP_3_ receptors activation. DHPG failed to increase fIPSPs in all CA1 **(a)** and specifically in its different sub-regions **(b–g)** in presence of the cell-permeable IP_3_ receptors blockers 2-APB or Xestospongin C (XestoC). In all figures, left panel shows the time course, while right panel is showing mean over time. Statistical analysis in right panel figures is one-way ANOVA followed by Dunnet *post-hoc* test. Vehicles of both IP_3_ receptors antagonists have been pooled together since they are not different. n = (slices, mice): Vehicle group = (11, 9), 2-APB group = (5, 5), Xestospongin C group (5, 3). Data are mean ± s.e.m. *p < 0.05, **p < 0.01, ***p < 0.001, ns = not significant.

Activation of mGlu_5_ triggers G_q_ protein signaling, which, *via* the inositol 1,4,5-trisphosphate cascade, ultimately leads to the recruitment of the ligand-gated Ca^2+^ release channels IP_3_ receptors in the endoplasmic reticulum (ER) and the increase of cytosolic Ca^2+ ^26,41. Therefore, we asked whether IP_3_ receptors are involved in the DHPG-induced potentiation of fIPSPs in the CA1 hippocampal region. Application of DHPG in continuous presence of the membrane permeable IP_3_ receptor antagonists 2-APB or Xestospongin C failed to increase VSDI-recorded hyperpolarization in the whole CA1 and in all the sub-regions analyzed (Fig. 6a–g), clearly pointing to the involvement of intracellular IP_3_ receptors in this effect.

## Discussion

This study shows that VDSI is a suitable technology to investigate network inhibitory activity in hippocampal slices, providing an equivalent of “field inhibitory postsynaptic potentials”, which depend on neuronal activity and are inhibited or potentiated by antagonism or allosteric enhancement of GABA_A_ receptors, respectively. As compared to classical electrophysiological techniques, a clear advantage of this approach is that it preserves spatial information, enabling the opportunity to dissect the intensity and distribution of fIPSPs amongst different sub-regions of a given brain area. Like other techniques, VSDI presents also specific drawbacks, such as the relative low levels of signal-to-noise ratio^19^,^20^, which is particularly evident when inhibitory field potentials are observed. However, the fact that the hyperpolarization observed in our study is fully blocked by TTX and Picrotoxin, and it is slightly but significantly increased by Chlordiazepoxide, clearly indicate the specific neuronal and GABA_A_ receptor-dependent nature of the signal observed. We took advantage of these properties to highlight a novel form of inhibitory synaptic plasticity, characterized by a long-lasting increase of GABAergic strength following mGlu_5_ and IP_3_ receptors activation.

The remarkable heterogeneity of CA1 hippocampal interneurons in terms of morphology and electrophysiological properties together with the extensive functional coupling to pyramidal cells^4^,^7^, underline the importance of monitoring GABAergic inhibitory activity at different neuro-architectural levels, from single cells to local circuits. Single cell recordings are valuable tools because of their ability to uncover sub-cellular input-output relationships and plasticity processes, but these approaches intrinsically lack the possibility to detect inhibitory transmission at larger network level, which can only be extrapolated, but not directly observed, from the data obtained. Very few attempts have been made to record network GABAergic activity^16^,^17^,^18^. In all these studies, single or few recording electrodes were used, thereby limiting the spatial information obtained about the GABAergic activity at network level.

In this context, our data reveal the possibility to study network GABAergic activity in large brain regions.

The presence of blockers of ionotropic glutamatergic transmission excludes synaptic activation of interneurons by glutamate released after Schaffer’s collaterals stimulation, and suggests that the observed phenomenon is likely mediated by direct recruitment of interneurons, leading to synchronous release of GABA in an action potential dependent manner. Indeed, this is strengthened by the fact that minimal stimulation intensity is sufficient to engage significant interneuron population.

In the mature brain GABA, by acting on ionotropic GABA_A_ receptors, inhibits excitation *via* two main mechanisms: hyperpolarization and shunting inhibition^1^. In our VSDI experiments, only hyperpolarization can be observed. Recent data, however, suggest that in the CA1 hippocampal region the hyperpolarizing component of GABA_A_ receptor activity might be preponderant^10^. Nevertheless, we cannot exclude the presence of GABA_A_ receptor shunting inhibition, which depends on the membrane potential state. These undetected events, however, would cause an underestimation of the GABAergic activity observed by VSDI recordings, further underlining the reliability of the approach.

Our data reveal a novel form of mGlu_5_-dependent plasticity of fIPSPs. In the hippocampal CA1 region, mGlu_1_ and mGlu_5_ are predominantly postsynaptic^42^. Whereas mGlu_1_ is mainly expressed in interneurons, and primarily those present in *alveus* and *stratum oriens*, mGlu_5_ is more widely present throughout the CA1, including the somatodendritic field of pyramidal cells, several classes of interneurons, and astrocytes^25^,^42^. Group-I mGlu receptors have a strong impact on neuronal activity by modulating cationic conductances, synaptic transmission and plasticity^27^. It is widely documented that activation of group-I mGlu receptors increases the excitability of hippocampal neurons^27^,^37^,^38^,^39^,^40^. In particular, mGlu_1_ activation has been shown to trigger a direct depolarization of pyramidal cells, while mGlu_5_ mediates a decrease of the slow after-hyperpolarization and a potentiation of NMDA currents^34^. Our data provide an additional effect to mGlu_5_ activation (potentiation of fIPSPs), which will have to be considered in further studies on group-I mGlu-mediated synaptic and plasticity events. The study of specific group-I mGlu receptors signaling on GABAergic activity in hippocampal CA1 has to date been restricted to single cell resolution^33^,^34^,^36^,^39^,^40^, with currently no data about network inhibitory activity. Interestingly, however, Gereau *et al*.^33^, showed that activation of group-I mGlu receptors by DHPG increases the frequency and not the amplitude of spontaneous IPSC recorded from pyramidal cells, suggesting an increase of GABA release by interneurons excited by the agonist. In addition, van Hooft and colleagues^36^ show that group-I mGlu receptors activation in several classes of *oriens-alveus* interneurons induces a dramatic increase of spike frequency and appearance of an inward current, consistent with group-I mGlu-induced increase of interneuron excitability. Furthermore, group-I mGlu receptor mediated increase in both interneuron excitability and/or GABA release, is not exclusive to the hippocampal CA1 region, but has also been reported in the thalamocortical neurons of dorsal lateral geniculate nucleus^43^, the ventral pallidum^44^, the periaqueductal grey^45^, retinal amacrine cells^46^, and in the entorhinal cortex^47^. These studies, however, show only a transient effect of DHPG on the electrophysiological activity of interneurons, which rapidly recovered to pre-drug conditions after washout of the compound. When we applied DHPG and recorded network GABA_A_ receptor-mediated activity in the CA1 using VSDI, we found that mGlu_5_ mediates a persistent enhancement of the signal that lasted for approximately 40–60 minutes after washout of the drug. Importantly, this effect is not attributable to a lack of drug washout, because the blockade of mGlu_5_ receptors after DHPG application still triggers long-lasting potentiation of fIPSPs. Therefore, our present data are in agreement with previous studies performed at single cell level and, in addition, show that the exciting effect of mGlu_5_ activation on inhibitory potentials induces phenomena of long-lasting plasticity when examined at network level. The differences in the duration of the stimulatory effects of mGlu_5_ on GABAergic functions between single cell studies and the here described network level may be ascribed to the perturbation of intracellular composition due to the patch clamp procedure. Indeed, this may be suggested by a recent paper^48^ demonstrating that mGlu receptors signaling in CA1 pyramidal cells is very sensitive to cytoplasmic dialysis, because using high resistance recording pipettes dramatically increases the amplitude and the duration of mGlu-mediated long-term depression of excitatory transmission. Future studies will investigate the role of intracellular dialysis in the short-to-long terms effects of mGlu_5_ signaling on GABA_A_ receptor activity.

The degree of the enhancement is region-specific inside the CA1, being generally more accentuated in the proximal part of *stratum radiatum* and in the region closer to the stimulation electrode, and weaker in the *stratum oriens* and in the pyramidal layer. The reasons for these differences are currently unknown. They could be related to the coincidence of interneuron activity in areas close to the stimulation, but they could also depend upon intrinsic differences between GABAergic network activities in different CA1 sub-fields. For instance, despite the fact that fIPSPs amplitude in the *stratum oriens* is comparable to other CA1 sub-layers, and that mGlu_5_ receptors are abundantly expressed in this sub-region^49^, this layer seems to be less sensitive to DHPG-induced potentiation of fIPSPs. Importantly, our data also show that the activation of IP_3_ receptors is a necessary step for mGlu_5_-induced potentiation of fIPSPs. Anatomical data^50^,^51^ indicate that IP_3_ receptors are less abundant in the *stratum oriens* than in other CA1 hippocampal layers (e.g. pyramidal layer and *stratum radiatum*), suggesting that the lower effect of DHPG in this sub-region might be due to the lower expression of key signaling elements downstream of mGlu_5_. The use of VSDI to study fIPSPs will allow future studies aimed at the precise anatomical, cellular, and molecular dissection of the plastic regulation of inhibitory transmission at the network level. For instance, given the growing body of literature suggesting that astrocytes are active regulators of GABAergic transmission^52^,^53^,^54^, it will be very interesting to address the role of these cell types in the regulation of fIPSPs.

Both mGlu_5_ and inhibitory transmission are involved in important central pathologies, such as, among others, epilepsy and Fragile X Syndrome^55^,^56^,^57^,^58^. The possibility to study GABAergic transmission at the network level provides an additional tool for a better understanding of brain function in physiological and pathological conditions. For instance, Deng and co-workers^47^, showed that high glutamate levels, such as in epilepsy, increase the frequency and amplitude of spontaneous IPSCs recorded on principal neurons of the entorhinal cortex, an effect that is mediated by mGlu_5_. In light of these results, we could speculate that in the case of intense glutamatergic activity, as occurs during a seizure, glutamate spillover may activate peri-synaptic mGlu_5_ receptors leading to a compensatory increase of network GABAergic activity. Interestingly, Campanac *et al*.^59^ shows a persistent increase of inhibitory potentials recorded in CA1 pyramidal neurons after high-frequency stimulation of Schaffer’s collaterals, an effect mediated by a long-term increase in the intrinsic excitability of parvalbumin-positive basket cells (PV^+^-BCs), and due to synaptic activation of mGlu_5_ receptors. It would be interesting to explore in future studies the role of PV^+^-BCs, and other interneurons populations, in the mGlu_5_-induced long-lasting potentiation reported here. Likewise, future experiments will address the physiological implications of these pharmacological results, by testing whether different inductions protocols triggering the release of endogenous glutamate might also induce similar effects as DHPG.

Collectively, our data show that VSDI allows the detection and quantification of *bona fide* inhibitory network activity, and highlight the tight neuromodulatory coupling of excitation and inhibition at mesoscale level.

## Methods

### Slice preparation and staining with voltage sensitive dye

Experiments were approved by and carried out according to the local ethical committee of the University of Bordeaux (approval number 501350-A) and the French Ministry of Agriculture and Forestry (authorization number 3306369).

8 to 11 weeks-old male C57BL/6-N mice (Janvier, France) were kept with *ad libitum* access to food and water, with 12 hours dark/light cycle (8 h00 pm/am).

Mice were decapitated after isoflurane anesthesia and 350 μm-thick sagittal slices containing dorsal hippocampus were cut with a vibratome (VT1200S, Leica, Germany).

During this procedure, the brain was immerged in ice-cold sucrose-based cutting solution bubbled with carbogen gas (95% O_2_/5% CO_2_) containing (in millimolar): 180 sucrose, 2.5 KCl, 26 NaHCO_3_, 1.25 NaH_2_PO_4_, 11 Glucose, 0.2 CaCl_2_, 12 MgCl_2_. After preparation, slices were transferred and incubated for 30 minutes at 34 °C in oxygenated artificial cerebrospinal fluid (ACSF) containing (in millimolar): 123 NaCl, 2.5 KCl, 26 NaHCO_3_, 1.25 NaH_2_PO_4_, 11 Glucose, 2,5 CaCl_2_, 1,3 MgCl_2_ and then allowed to recover at room temperature in the same solution for at least 30 minutes before the staining procedure with the dye. Each slice was stained for 15 minutes in ACSF, under continuous carbogen flow, with the voltage sensitive fluorescent dye Di-4-ANEPPS (Sigma-Aldrich, France) at a concentration of 16,4 μM in DMSO (DMSO <0.1%).

The stained slice was then left to recover for at least 45 minutes, in dye-free ACSF at room temperature, before recordings. Mennerick *et al*.^60^ found that Di-4-ANEPPS increases GABA_A_ receptor conductance which is associated with a decreased network spontaneous spiking activity in dissociated cultures of hippocampal neurons. However, the effects reported are completely reversible to baseline level after washout of the cultures with dye-free solution and therefore exclude the impact of Di-4-ANEPPS-induced modulation of GABAergic activity on our VSDI recordings.

### Optical recording method

Slices were placed in a recording chamber (Membrane Chamber; Scientific Systems Design Inc., Canada) under constant oxygenated ACSF flow (~2 ml/min) at room temperature.

To record neuronal signals with VSDI we used an epifluorescence macroscope (Brainvision, Japan) equipped with the MiCAM02 optical imaging system (MiCAM02–HR; Brainvision, Japan) with a spatial resolution of 33.3 × 37.5 μm (horizontal and vertical, respectively) for each pixel.

A stereoscopic microscope (Leica, Germany) was used to visually guide the stimulating concentric bipolar electrode (FHC Inc., USA, catalog number CBARC75) into the proximal (respect to CA3) part of *stratum radiatum* to activate the Schaffer’s collateral pathway. To improve the signal-to-noise ratio of the GABA_A_ receptor-mediated hyperpolarization, for all the experiments, we set stimulation intensity at the maximum of the Input-Output curve (20 Volts, Fig. 2a) with a duration of 200 μs each stimulus, using an isolated voltage stimulator (DS2A, Digitimer Ltd., United Kingdom).

One acquisition consisted of 256 frames sampled every 2.2 ms, averaged 15 times at a time interval of 5 seconds (acquisition duration is ~70 seconds).

In experiments with DHPG, we performed six acquisitions as baseline, we then applied DHPG for ten minutes and finally we performed thirteen acquisitions during washout of DHPG, with an acquisition interval of 4 minutes.

In all experiments, before application of blockers of ionotropic glutamatergic transmission, one acquisition was taken in drug-free ACSF to check for slice health.

### Data analysis

To quantify VSDI signals we calculated the fractional change in fluorescence (ΔF*F^−1^) and we spatially smoothed the ΔF*F^−1^ values with a 3 × 3 spatial filter using the image analysis-acquisition software (Brainvision, Japan). Exclusively for Supplementary Videos S1, S2 and S3, we used a spatial filter of 5 × 5 pixels, after ΔF*F^−1^ signal normalization. In Fig. 1a,d,g and Supplementary Videos S1, S2 and S3 we isolated the CA1 region with a region of interest (ROI) by zeroing smoothed ΔF*F^−1^ values outside the ROI.

A depolarization produces a reduction in fluorescence emitted by Di-4-ANEPPS, while a hyperpolarization an increase; therefore, for clarity, ΔF*F^−1^ values representing depolarization (Fig. 1b,e) were considered positive.

ROIs were *post-hoc* visually drawn onto the slice, according to the representative spatial arrangement as shown in Figs 1b,c and 3b,e, using the image analysis-acquisition software (Brainvision, Japan). All the possible has been done to exactly match the ROI boundaries with anatomical landmarks. However, the large spatial resolution of our VSDI recordings together with the relatively large size of each pixel, make it difficult to create an exact anatomical sub-division inside the CA1 region and therefore, the ROI named “Radt. Distal” (*radiatum* distal) contain *stratum lacunosum-moleculare* as well, while the ROI named “Pyr. Layer” (Pyramidal Layer) may include very limited parts of *stratum oriens* and *stratum radiatum*.

To draw ROIs in *stratum radiatum*, we first defined the ROI “Radt. Prox” and then we moved it ventrally at a position adjacent of the previous one to obtain the ROI “Radt. Dist”. To design ROIs along the proximo-distal axis of the CA1 relative to the stimulation electrode (named “P = proximal”, “M = medial” and “D = distal), we first drew the ROI “P”, which was then duplicated and moved distally at adjacent points to obtain the ROIs “M” and then “D”. For the quantification of hyperpolarization signal across the sub-regions of CA1 (Fig. 3; schematic representation in b and e), we did as follows: at the middle of each dorso-ventral ROI (“str. Oriens”, “Pyr. Layer”, “Radt. Prox” and “Radt. Dist”) we drew a line (1 pixel wide, 8 pixels long) starting from the initial boundary relative to the stimulation electrode position. The same eight pixels long line was then positioned in the middle of each proximo-distal ROI (“P”, “M”, and “D”). To measure lengths along the proximo-distal axis of CA1 (Fig. 3f,g) we considered a pixel as a square of 35.4 μm side, resulting from the mean of actual pixel size. A summary of all ROI sizes for experiments in Figs 1f, 2 and 4, 5, 6 and Supplementary Figs S1, S2 and S3 is available in the Supplementary Table S1.

To quantify GABA_A_ receptor-mediated hyperpolarization recorded with VSDI we calculated the Area Under Curve (AUC) of traces representing mean ΔF*F^−1^ values over time of each ROI, using a time interval of 200 milliseconds, starting from the time of hyperpolarization appearance (approximately 5 milliseconds after stimulus onset). To quantify depolarization signals in the presence of Picrotoxin (Fig. 1f and Supplementary Fig. S1), we calculated AUC values considering a time window of 30 milliseconds starting from the time point before stimulation. AUCs quantifications were performed with Axograph X (version 1.5, Axograph, USA).

Background AUC values (Figs 2b–e and 3c,d,f,g; Supplementary Fig. S2) were calculated measuring the AUC of traces from the same ROIs used for evaluation of signal of interest, which have been moved outside the hippocampus (in either cortex or thalamus). Final AUC values of background are the mean of three ROIs (except in Fig. 2b–e and Supplementary Fig. S2 where they are the mean of three ROIs for baseline and drug application, respectively).

In experiments with DHPG, “Baseline” is the mean of AUC values calculated for each ROI from the last four acquisitions before DHPG application. Except time “zero”, all others time points after DHPG are the mean of the AUC from four subsequent acquisitions. Data are then represented as percentage variation of mean AUC values with respect to mean baseline.

### Pharmacology

2,3-Dioxo-6-nitro-1,2,3,4-tetrahydrobenzo[f]quinoxaline-7-sulfonamide disodium salt (NBQX), D-(-)-2-Amino-5-phosphonopentanoic acid (APV), (S)-3,5-Dihydroxyphenylglycine (DHPG), (S)-(+)-α-Amino-4-carboxy-2-methylbenzeneacetic acid (LY367385), 2-Methyl-6-(phenylethynyl)pyridine hydrochloride (MPEP) were purchased from Abcam (France). Stock solutions of NBQX, D-APV, DHPG and MPEP were made in water, while LY367385 was dissolved in 100 mM of NaOH (final NaOH was ~0.1%). Once aliquoted, DHPG was used within one week. Slices were incubated with mGlu receptors antagonists from the start of the post-staining recovery period and, unless stated otherwise, until the end of DHPG application.

Octahydro-12-(hydroxymethyl)-2-imino-5,9:7,10a-dimethano-10aH-1,3]dioxocino[6,5-d]pyrimidine-4,7,10,11,12-pentol citrate (TTX), (2S)-3-[[(1S)-1-(3,4-Dichlorophenyl)ethyl]amino-2-hydroxypropyl](phenylmethyl)phosphinic acid hydrochloride (CGP55845) and 2-Aminoethoxydiphenylborane (2-APB) were purchased from Tocris (United Kingdom). TTX was dissolved in water, while CGP55845 and 2-APB in DMSO (DMSO <0.1%). Xestospongin C was from Enzo Life Sciences (France) and dissolved in DMSO (DMSO <0.1%).

Picrotoxin (PTX) and 7-Chloro-2-(methylamino)-5-phenyl-3H-1,4-benzodiazepine 4-oxide hydrochloride (Chlordiazepoxide, CDP) were from Sigma-Aldrich (France). CDP was dissolved in water, while PTX in 100% ethanol. Drugs were bath-applied.

NBQX, APV and TTX were applied for 15 minutes, whereas PTX, CDP, and CGP55845 were applied for 30 minutes.

### Statistics

Data are expressed as mean ± s.e.m. All graphs and statistical analyses were performed with GraphPad Prism software (version 6.0). Two-tailed paired or unpaired t-test, one-way ANOVA, one-way repeated measures ANOVA, or two-way ANOVA followed by Tukey, Bonferroni or Dunnett *post-hoc* tests were used as appropriate. Differences were considered significant if p < 0.05.

## Additional Information

**How to cite this article**: Colavita, M. *et al*. Layer-specific potentiation of network GABAergic inhibition in the CA1 area of the hippocampus. *Sci. Rep.* **6**, 28454; doi: 10.1038/srep28454 (2016).

## Supplementary Material

Supplementary Information

Supplementary Video 1

Supplementary Video 2

Supplementary Video 3

## References

[b1] Fishell G. & Rudy B. Mechanisms of inhibition within the telencephalon: “where the wild things are”. Annu Rev Neurosci 34, 535–67 (2011). 21469958 10.1146/annurev-neuro-061010-113717 PMC3556485

[b2] Bezaire M. J. & Soltesz I. Quantitative assessment of CA1 local circuits: knowledge base for interneuron-pyramidal cell connectivity. Hippocampus 23, 751–85 (2013). 23674373 10.1002/hipo.22141 PMC3775914

[b3] Aika Y., Ren J. Q., Kosaka K. & Kosaka T. Quantitative analysis of GABA-like-immunoreactive and parvalbumin-containing neurons in the CA1 region of the rat hippocampus using a stereological method, the disector. Exp Brain Res 99, 267–76 (1994). 7925807 10.1007/BF00239593

[b4] Freund T. F. & Buzsaki G. Interneurons of the hippocampus. Hippocampus 6, 347–470 (1996). 8915675 10.1002/(SICI)1098-1063(1996)6:4<347::AID-HIPO1>3.0.CO;2-I

[b5] Chamberland S. & Topolnik L. Inhibitory control of hippocampal inhibitory neurons. Front Neurosci 6, 165 (2012). 23162426 10.3389/fnins.2012.00165 PMC3496901

[b6] Klausberger T. & Somogyi P. Neuronal diversity and temporal dynamics: the unity of hippocampal circuit operations. Science 321, 53–7 (2008). 18599766 10.1126/science.1149381 PMC4487503

[b7] Petilla Interneuron Nomenclature G. . Petilla terminology: nomenclature of features of GABAergic interneurons of the cerebral cortex. Nat Rev Neurosci 9, 557–68 (2008). 18568015 10.1038/nrn2402 PMC2868386

[b8] Isaacson J. S. & Scanziani M. How inhibition shapes cortical activity. Neuron 72, 231–43 (2011). 22017986 10.1016/j.neuron.2011.09.027 PMC3236361

[b9] Kullmann D. M. Interneuron networks in the hippocampus. Curr Opin Neurobiol 21, 709–16 (2011). 21636266 10.1016/j.conb.2011.05.006

[b10] Glickfeld L. L., Roberts J. D., Somogyi P. & Scanziani M. Interneurons hyperpolarize pyramidal cells along their entire somatodendritic axis. Nat Neurosci 12, 21–3 (2009). 19029887 10.1038/nn.2230 PMC3505023

[b11] Buzsaki G. Feed-forward inhibition in the hippocampal formation. Prog Neurobiol 22, 131–53 (1984). 6433403 10.1016/0301-0082(84)90023-6

[b12] Freund T. F. & Katona I. Perisomatic inhibition. Neuron 56, 33–42 (2007). 17920013 10.1016/j.neuron.2007.09.012

[b13] Pouille F. & Scanziani M. Enforcement of temporal fidelity in pyramidal cells by somatic feed-forward inhibition. Science 293, 1159–63 (2001). 11498596 10.1126/science.1060342

[b14] Cobb S. R. . Synaptic effects of identified interneurons innervating both interneurons and pyramidal cells in the rat hippocampus. Neuroscience 79, 629–48 (1997). 9219929 10.1016/s0306-4522(97)00055-9

[b15] Sik A., Penttonen M., Ylinen A. & Buzsaki G. Hippocampal CA1 interneurons: an *in vivo* intracellular labeling study. J Neurosci 15, 6651–65 (1995). 7472426 10.1523/JNEUROSCI.15-10-06651.1995 PMC6577981

[b16] Arai A., Silberg J. & Lynch G. Differences in the refractory properties of two distinct inhibitory circuitries in field CA1 of the hippocampus. Brain Res 704, 298–306 (1995). 8788926 10.1016/0006-8993(95)01137-4

[b17] Bazelot M., Dinocourt C., Cohen I. & Miles R. Unitary inhibitory field potentials in the CA3 region of rat hippocampus. J Physiol 588, 2077–90 (2010). 20403979 10.1113/jphysiol.2009.185918 PMC2911213

[b18] Lambert N. A., Borroni A. M., Grover L. M. & Teyler T. J. Hyperpolarizing and depolarizing GABAA receptor-mediated dendritic inhibition in area CA1 of the rat hippocampus. J Neurophysiol 66, 1538–48 (1991). 1684989 10.1152/jn.1991.66.5.1538

[b19] Chemla S. & Chavane F. Voltage-sensitive dye imaging: Technique review and models. J Physiol Paris 104, 40–50 (2010). 19909809 10.1016/j.jphysparis.2009.11.009

[b20] Peterka D. S., Takahashi H. & Yuste R. Imaging voltage in neurons. Neuron 69, 9–21 (2011). 21220095 10.1016/j.neuron.2010.12.010 PMC3387979

[b21] Loew L. M. . A naphthyl analog of the aminostyryl pyridinium class of potentiometric membrane dyes shows consistent sensitivity in a variety of tissue, cell, and model membrane preparations. J Membr Biol 130, 1–10 (1992). 1469705 10.1007/BF00233734

[b22] Grinvald A., Manker A. & Segal M. Visualization of the spread of electrical activity in rat hippocampal slices by voltage-sensitive optical probes. J Physiol 333, 269–91 (1982). 7182467 10.1113/jphysiol.1982.sp014453 PMC1197248

[b23] Tominaga T., Tominaga Y., Yamada H., Matsumoto G. & Ichikawa M. Quantification of optical signals with electrophysiological signals in neural activities of Di-4-ANEPPS stained rat hippocampal slices. J Neurosci Methods 102, 11–23 (2000). 11000407 10.1016/s0165-0270(00)00270-3

[b24] Canepari M., Willadt S., Zecevic D. & Vogt K. E. Imaging inhibitory synaptic potentials using voltage sensitive dyes. Biophys J 98, 2032–40 (2010). 20441768 10.1016/j.bpj.2010.01.024 PMC2862202

[b25] Ferraguti F., Crepaldi L. & Nicoletti F. Metabotropic glutamate 1 receptor: current concepts and perspectives. Pharmacol Rev 60, 536–81 (2008). 19112153 10.1124/pr.108.000166

[b26] Fagni L., Chavis P., Ango F. & Bockaert J. Complex interactions between mGluRs, intracellular Ca^2+^ stores and ion channels in neurons. Trends Neurosci 23, 80–8 (2000). 10652549 10.1016/s0166-2236(99)01492-7

[b27] Anwyl R. Metabotropic glutamate receptors: electrophysiological properties and role in plasticity. Brain Res Brain Res Rev 29, 83–120 (1999). 9974152 10.1016/s0165-0173(98)00050-2

[b28] Castillo P. E., Chiu C. Q. & Carroll R. C. Long-term plasticity at inhibitory synapses. Curr Opin Neurobiol 21, 328–38 (2011). 21334194 10.1016/j.conb.2011.01.006 PMC3092861

[b29] Mann E. O., Tominaga T., Ichikawa M. & Greenfield S. A. Cholinergic modulation of the spatiotemporal pattern of hippocampal activity *in vitro*. Neuropharmacology 48, 118–33 (2005). 15617733 10.1016/j.neuropharm.2004.08.022

[b30] Megias M., Emri Z., Freund T. F. & Gulyas A. I. Total number and distribution of inhibitory and excitatory synapses on hippocampal CA1 pyramidal cells. Neuroscience 102, 527–40 (2001). 11226691 10.1016/s0306-4522(00)00496-6

[b31] Davies C. H., Davies S. N. & Collingridge G. L. Paired-pulse depression of monosynaptic GABA-mediated inhibitory postsynaptic responses in rat hippocampus. J Physiol 424, 513–31 (1990). 2167975 10.1113/jphysiol.1990.sp018080 PMC1189826

[b32] Luscher C. & Huber K. M. Group 1 mGluR-dependent synaptic long-term depression: mechanisms and implications for circuitry and disease. Neuron 65, 445–59 (2010). 20188650 10.1016/j.neuron.2010.01.016 PMC2841961

[b33] Gereau R. W. t. & Conn P. J. Multiple presynaptic metabotropic glutamate receptors modulate excitatory and inhibitory synaptic transmission in hippocampal area CA1. J Neurosci 15, 6879–89 (1995). 7472445 10.1523/JNEUROSCI.15-10-06879.1995 PMC6578030

[b34] Mannaioni G., Marino M. J., Valenti O., Traynelis S. F. & Conn P. J. Metabotropic glutamate receptors 1 and 5 differentially regulate CA1 pyramidal cell function. J Neurosci 21, 5925–34 (2001). 11487615 10.1523/JNEUROSCI.21-16-05925.2001 PMC6763150

[b35] McBain C. J., DiChiara T. J. & Kauer J. A. Activation of metabotropic glutamate receptors differentially affects two classes of hippocampal interneurons and potentiates excitatory synaptic transmission. J Neurosci 14, 4433–45 (1994). 7517996 10.1523/JNEUROSCI.14-07-04433.1994 PMC6577047

[b36] van Hooft J. A., Giuffrida R., Blatow M. & Monyer H. Differential expression of group I metabotropic glutamate receptors in functionally distinct hippocampal interneurons. J Neurosci 20, 3544–51 (2000). 10804195 10.1523/JNEUROSCI.20-10-03544.2000 PMC6772706

[b37] Miles R. & Poncer J. C. Metabotropic glutamate receptors mediate a post-tetanic excitation of guinea-pig hippocampal inhibitory neurones. J Physiol 463, 461–73 (1993). 7902437 10.1113/jphysiol.1993.sp019605 PMC1175354

[b38] Poncer J. C., Shinozaki H. & Miles R. Dual modulation of synaptic inhibition by distinct metabotropic glutamate receptors in the rat hippocampus. J Physiol 485 (Pt 1), 121–34 (1995). 7658367 10.1113/jphysiol.1995.sp020717 PMC1157977

[b39] Gee C. E. & Lacaille J. C. Group I metabotropic glutamate receptor actions in oriens/alveus interneurons of rat hippocampal CA1 region. Brain Res 1000, 92–101 (2004). 15053957 10.1016/j.brainres.2003.11.046

[b40] Woodhall G., Gee C. E., Robitaille R. & Lacaille J. C. Membrane potential and intracellular Ca^2+^ oscillations activated by mGluRs in hippocampal stratum oriens/alveus interneurons. J Neurophysiol 81, 371–82 (1999). 9914296 10.1152/jn.1999.81.1.371

[b41] Foskett J. K., White C., Cheung K. H. & Mak D. O. Inositol trisphosphate receptor Ca^2+^ release channels. Physiol Rev 87, 593–658 (2007). 17429043 10.1152/physrev.00035.2006 PMC2901638

[b42] Ferraguti F. & Shigemoto R. Metabotropic glutamate receptors. Cell Tissue Res 326, 483–504 (2006). 16847639 10.1007/s00441-006-0266-5

[b43] Errington A. C., Di Giovanni G., Crunelli V. & Cope D. W. mGluR control of interneuron output regulates feedforward tonic GABAA inhibition in the visual thalamus. J Neurosci 31, 8669–80 (2011). 21653871 10.1523/JNEUROSCI.0317-11.2011 PMC3130900

[b44] Diaz-Cabiale Z. . Metabotropic glutamate mGlu5 receptor-mediated modulation of the ventral striopallidal GABA pathway in rats. Interactions with adenosine A(2A) and dopamine D(2) receptors. Neurosci Lett 324, 154–8 (2002). 11988350 10.1016/s0304-3940(02)00179-9

[b45] de Novellis V. . Group I metabotropic glutamate receptors modulate glutamate and gamma-aminobutyric acid release in the periaqueductal grey of rats. Eur J Pharmacol 462, 73–81 (2003). 12591098 10.1016/s0014-2999(03)01342-6

[b46] Hoffpauir B. K. & Gleason E. L. Activation of mGluR5 modulates GABA(A) receptor function in retinal amacrine cells. J Neurophysiol 88, 1766–76 (2002). 12364505 10.1152/jn.2002.88.4.1766

[b47] Deng P. Y., Xiao Z. & Lei S. Distinct modes of modulation of GABAergic transmission by Group I metabotropic glutamate receptors in rat entorhinal cortex. Hippocampus 20, 980–93 (2010). 19739246 10.1002/hipo.20697 PMC2891270

[b48] Fan W., Ster J. & Gerber U. Activation conditions for the induction of metabotropic glutamate receptor-dependent long-term depression in hippocampal CA1 pyramidal cells. J Neurosci 30, 1471–5 (2010). 20107074 10.1523/JNEUROSCI.5619-09.2010 PMC6633811

[b49] Smialowska M. . Effect of chronic imipramine or electroconvulsive shock on the expression of mGluR1a and mGluR5a immunoreactivity in rat brain hippocampus. Neuropharmacology 42, 1016–23 (2002). 12128002 10.1016/s0028-3908(02)00062-x

[b50] Hertle D. N. & Yeckel M. F. Distribution of inositol-1,4,5-trisphosphate receptor isotypes and ryanodine receptor isotypes during maturation of the rat hippocampus. Neuroscience 150, 625–38 (2007). 17981403 10.1016/j.neuroscience.2007.09.058 PMC2238340

[b51] Pieper A. A. . Differential neuronal localizations and dynamics of phosphorylated and unphosphorylated type 1 inositol 1,4,5-trisphosphate receptors. Neuroscience 102, 433–44 (2001). 11166129 10.1016/s0306-4522(00)00470-x

[b52] Christian C. A. & Huguenard J. R. Astrocytes potentiate GABAergic transmission in the thalamic reticular nucleus via endozepine signaling. Proc Natl Acad Sci USA 110, 20278–83 (2013). 24262146 10.1073/pnas.1318031110 PMC3864346

[b53] Jo S. . GABA from reactive astrocytes impairs memory in mouse models of Alzheimer’s disease. Nat Med 20, 886–96 (2014). 24973918 10.1038/nm.3639 PMC8385452

[b54] Yoon B. E. . Glial GABA, synthesized by monoamine oxidase B, mediates tonic inhibition. J Physiol 592, 4951–68 (2014). 25239459 10.1113/jphysiol.2014.278754 PMC4259537

[b55] Piers T. M. . Translational Concepts of mGluR5 in Synaptic Diseases of the Brain. Front Pharmacol 3, 199 (2012). 23205012 10.3389/fphar.2012.00199 PMC3506921

[b56] Bear M. F., Huber K. M. & Warren S. T. The mGluR theory of fragile X mental retardation. Trends Neurosci 27, 370–7 (2004). 15219735 10.1016/j.tins.2004.04.009

[b57] D’Hulst C. & Kooy R. F. The GABAA receptor: a novel target for treatment of fragile X? Trends Neurosci 30, 425–31 (2007). 17590448 10.1016/j.tins.2007.06.003

[b58] Treiman D. M. GABAergic mechanisms in epilepsy. Epilepsia 42 Suppl 3, 8–12 (2001). 11520315 10.1046/j.1528-1157.2001.042suppl.3008.x

[b59] Campanac E. . Enhanced intrinsic excitability in basket cells maintains excitatory-inhibitory balance in hippocampal circuits. Neuron 77, 712–22 (2013). 23439123 10.1016/j.neuron.2012.12.020

[b60] Mennerick S. . Diverse voltage-sensitive dyes modulate GABAA receptor function. J Neurosci 30, 2871–9 (2010). 20181584 10.1523/JNEUROSCI.5607-09.2010 PMC2840391

