# Supplementary material for: Layer-specific potentiation of network GABAergic inhibition in the CA1 area of the hippocampus
Source: Sci Rep. 2016 Jun 27;6:28454. doi: 10.1038/srep28454 (PMC4921906; doi:10.1038/srep28454)
Supplement: Supplementary Information [file srep28454-s1.pdf]

Supplementary information

Layer-specific potentiation of network GABAergic inhibition in the CA1 area of the hippocampus

Michelangelo Colavita, Geoffrey Terral, Clement E. Lemercier, Filippo Drago, Giovanni Marsicano, Federico Massa

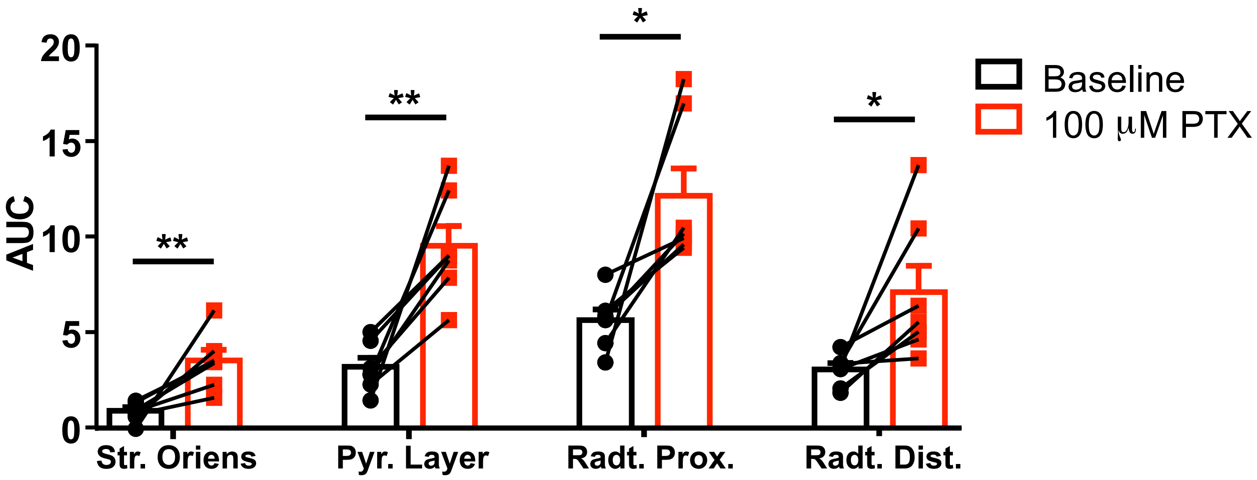

Supplementary Figure S1. Impact of blockade of GABAergic transmission on VSD-recorded depolarization in specific CA1 sub-regions. Application of the $GABA_A$ receptor antagonist Picrotoxin (PTX, 100 μM) significantly increases VSD-recorded depolarization in all the CA1 sub-regions. n = 7 slices from 5 mice. Statistical significance has been assessed with two-tailed paired *t*-test. Data in bars are mean ± s.e.m. whereas over imposed lines are single values. * = $p<0.05$, ** = $p<0.01$.

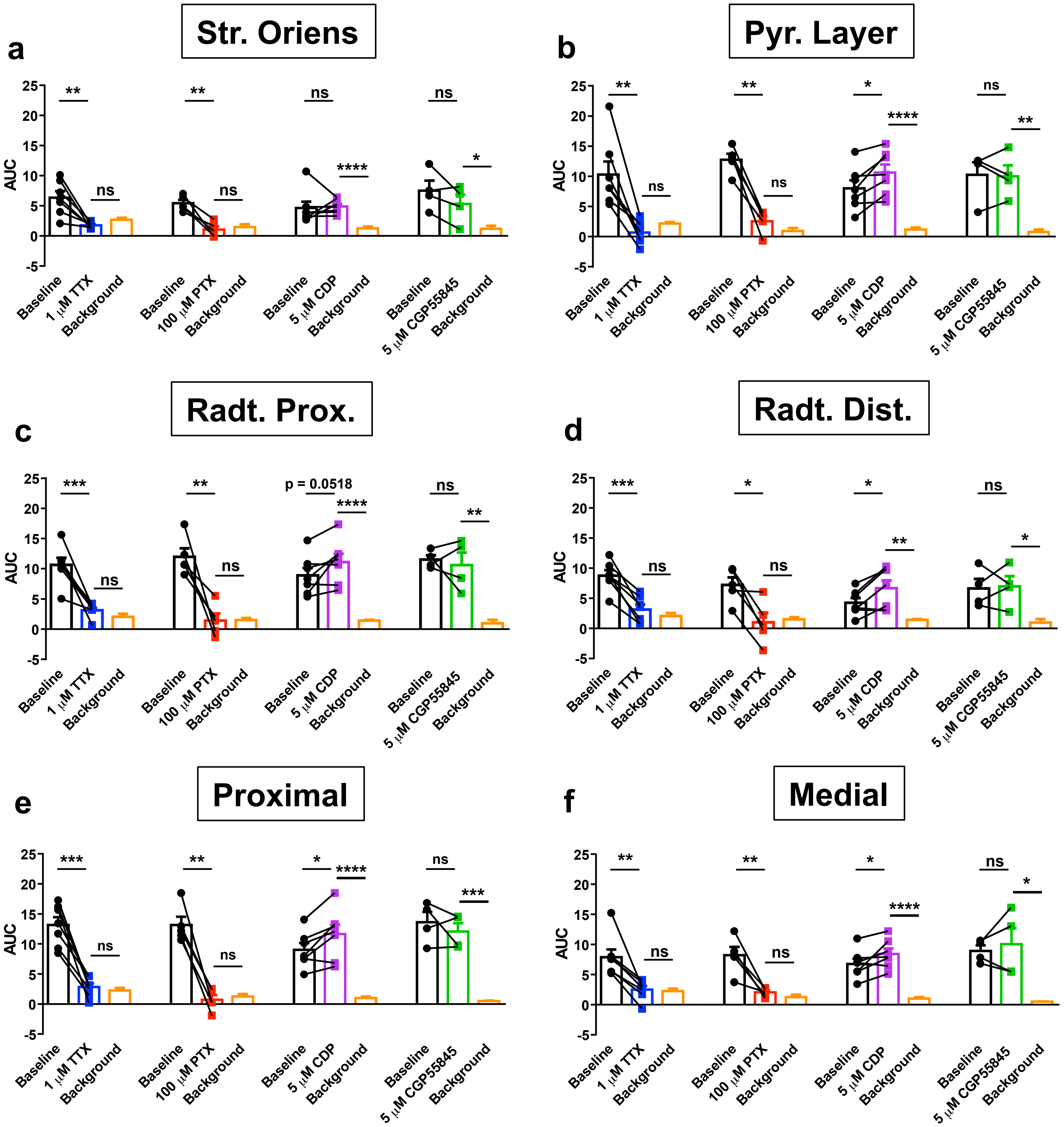

Supplementary Figure S2. Region-specific pharmacological characterization of VSD-recorded fIPSPs in CA1 region. Tetrodotoxin (TTX, 1 μM) = 7 slices from 5 mice; Picrotoxin (PTX, 100 μM) 5 slices from 3 mice ; Chlordiazepoxide (CDP, 5 μM) = 7 slices from 4 mice; CGP55845 (5 μM) = 4 slices from 2 mice. Data in bars are mean ± s.e.m. whereas over imposed lines are single values. Statistical significance has been assessed with two-tailed paired *t*-test between baseline condition and drug application, while two-tailed unpaired *t*-test has been used between drug application and respective Background. * = $p<0.05$, ** = $p<0.01$, *** = $p<0.001$, **** = $p<0.0001$, ns = not significant.

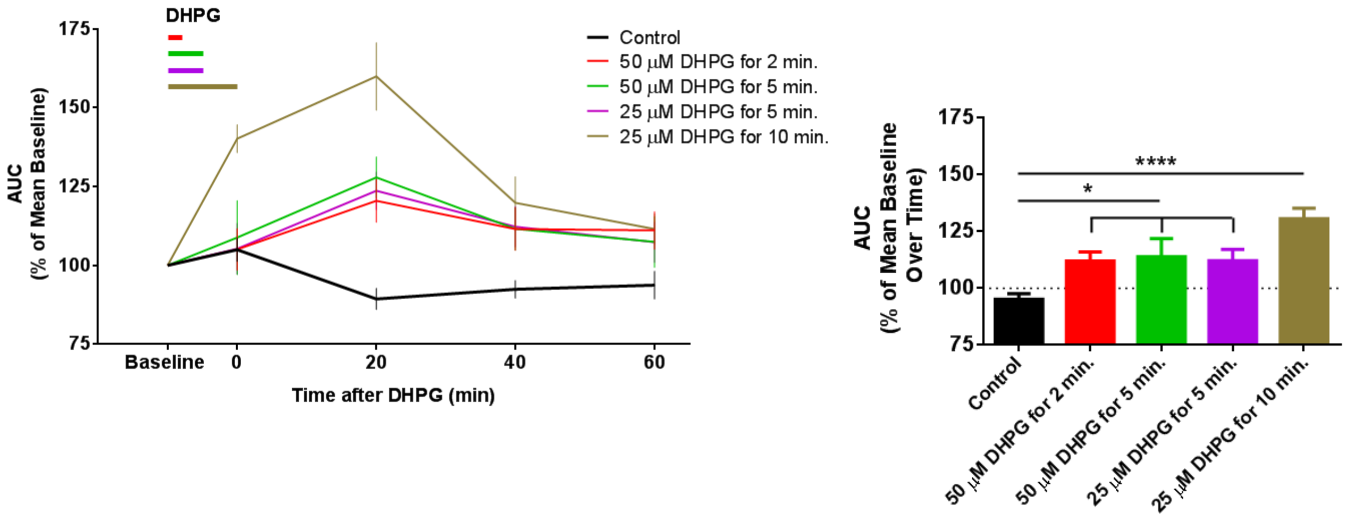

Supplementary Figure S3. Different doses and durations of DHPG application increase VSD-recorded fIPSPs in the whole CA1 region. Application of DHPG at indicated doses and times significantly increases VSD-recorded fIPSPs respect to control condition, where no DHPG has been added. Data are from a ROI covering the whole CA1. Left panel shows time course, while right panel is mean over time for each condition. n = (slices, mice) for each group: Control (7,4), 50 µM DHPG for 2 minutes (4,2), 50 µM DHPG for 5 minutes (4,3), 25 µM DHPG for 5 minutes (5,2), 25 µM DHPG for 10 minutes (5,3). Data are mean ± s.e.m. Statistical test in right panel is one-way ANOVA ($F_{(4, 20)}$ = 8.779, p=0.0003) followed by Dunnet *post-hoc* test. * = p<0.05, **** = p<0.0001.

Supplementary Table 1

Experiment in Figure n°	Mean ± standard deviation (number of pixels); number of slices						
	Whole CA1	Str. Oriens	Pyramidal Layer	*radiatum* proximal	*radiatum* distal	Proximal region	Medial region
**1f and Supplementary Figure 1**	317 ± 8; 7	135 ± 21; 7	37 ± 7; 7	82 ± 5; 7	82 ± 5; 7	106 ± 11; 7	106 ± 11; 7
**2a**	311 ± 14; 6						
**2b** and **Supplementary Figure 2**	308 ± 14; 7	112 ± 16; 7	26 ± 9; 7	62 ± 12; 7	62 ± 12; 7	85 ± 10; 7	85 ± 10; 7
**2c** and **Supplementary Figure 2**	317 ± 14; 5	119 ± 13; 5	23 ± 3; 5	64 ± 8; 5	64 ± 8; 5	84 ± 13; 5	84 ± 13; 5
**2d** and **Supplementary Figure 2**	300 ± 18; 4	133 ± 7; 4	24 ± 4; 4	76 ± 7; 4	76 ± 7; 4	93 ± 16; 4	93 ± 16; 4
**2e** and **Supplementary Figure 2**	322 ± 15; 7	124 ± 12; 7	27 ± 6; 7	63 ± 8; 7	63 ± 8; 7	89 ± 10; 7	89 ± 10; 7
**4 - DHPG**	311 ± 15; 7	124 ± 22; 7	25 ± 7; 7	61 ± 10; 7	61 ± 10; 7	74 ± 9; 7	74 ± 9; 7
**4 - Control**	300 ± 17; 7	144 ± 14; 7	32 ± 11; 7	67 ± 5; 7	67 ± 5; 7	72 ± 13; 7	72 ± 13; 7
**5 - Vehicle**	310 ± 13; 7	123 ± 13; 7	25 ± 5; 7	65 ± 10; 7	65 ± 10; 7	77 ± 8; 7	77 ± 8; 7
**5 - LY367385 + MPEP Before**	311 ± 17; 7	130 ± 20; 7	25 ± 6; 7	60 ± 11; 7	60 ± 11; 7	75 ± 14; 7	75 ± 14; 7
**5 - MPEP Before**	299 ± 14; 6	146 ± 27; 6	26 ± 9; 6	67 ± 5; 6	67 ± 5; 6	83 ± 15; 6	83 ± 15; 6
**5 - MPEP After**	311 ± 11; 5	107 ± 20; 5	36 ± 5; 5	62 ± 9; 5	62 ± 9; 5	85 ± 13; 5	85 ± 13; 5
**5 - LY367385**	308 ± 12; 7	128 ± 19; 7	22 ± 5; 7	60 ± 12; 7	60 ± 12; 7	85 ± 7; 7	85 ± 7; 7
**6 - Vehicle**	306 ± 15; 11	128 ± 14; 11	26 ± 5; 11	66 ± 8; 11	66 ± 8; 11	93 ± 13; 11	93 ± 13; 11
**6 - 2-APB**	298 ± 10; 5	117 ± 20; 5	33 ± 6; 5	62 ± 10; 5	62 ± 10; 5	86 ± 11; 5	86 ± 11; 5
**6 - Xestospongin C**	315 ± 21; 5	107 ± 9; 5	24 ± 3; 5	74 ± 9; 5	74 ± 9; 5	97 ± 14; 5	97 ± 14; 5
**Supplementary Figure 3 - Control**	300 ± 17; 7						
**Supplementary Figure 3 - 50 µM DHPG for 2 min.**	307 ± 11; 4						
**Supplementary Figure 3 - 50 µM DHPG for 5 min.**	315 ± 12; 4						
**Supplementary Figure 3 - 25 µM DHPG for 5 min.**	325 ± 8; 5						
**Supplementary Figure 3 - 25 µM DHPG for 10 min.**	304 ± 7; 5						

Captions for Supplementary Videos:

Supplementary Video V1: Voltage sensitive dye (VSD) – recorded depolarization along CA1 region in drug-free ACSF.

Supplementary Video V2: VSD – recorded depolarization along CA1 region in presence of the $GABA_A$ receptor antagonist Picrotoxin (PTX, 100 μM).

Supplementary Video V3: VSD – recorded hyperpolarization along CA1 region in presence of the AMPA/Kainate and NMDA receptors antagonists NBQX (10 μM) and APV (50 μM), respectively.

In all videos time - lapse is shown on the bottom right (ms = milliseconds; frames interleaved of 2.2 milliseconds).
